# Psychometric properties and validation of the metacognitive self-assessment scale (MSAS) in a Turkish sample

**DOI:** 10.3389/fpsyg.2024.1411733

**Published:** 2024-12-12

**Authors:** Eda Yılmazer, Zeynep Hamamcı, Fulya Türk

**Affiliations:** ^1^Psychology Department, Faculty of Social Sciences, Beykoz University, Istanbul, Türkiye; ^2^Department of Guidance and Psychological Counseling, Faculty of Education, Yildiz Technical University, Istanbul, Türkiye

**Keywords:** metacognition, assessment, reliability, validity, scale

## Abstract

**Objective:**

Metacognition, a multifaceted psychological construct, encompasses recognising and explaining one’s cognitive processes and those of others. Notably, deficits in metacognitive abilities are linked with diminished social performance, reduced quality of life, and increased severity of Personality Disorders (PD). While there are other assessment tools available in Turkish for evaluating metacognition, none offer the same combination of speed, simplicity, flexibility, and multidimensionality for screening metacognitive abilities as the Metacognition Self-Assessment Scale (MSAS).The Metacognitive Self-Assessment Scale (MSAS) was designed to evaluate various metacognitive functions—namely, Monitoring, Integration, Differentiation, and Disintegration—as described by the Metacognitive Multifunction Model. This study aims to translate and validate the MSAS for Turkish culture.

**Method:**

To check the factor structure’s suitability for the Turkish population, 467 non-clinical participants (67.7% female, ranged from 18 to 31, mean 24.18; ±3.25) were included.

**Results:**

Initial analyses confirm that the scale is a valid and dependable instrument for Turkish culture. Construct validity, represented by the 18 items and four subscales, was confirmed through Confirmatory Factor Analysis (CFA) and criterion validity assessments. As well as the test–retest reliability of the scale was confirmed. Based on these findings, it was concluded that the scale is valid and reliable for use in Turkey. The model aligns well with empirical data, highlighting its strong construct validity and indicating good reliability of the scale.

**Conclusion:**

The Turkish version of the MSAS, developed to measure metacognition and its associated components, has proven to be a credible and reliable tool, especially in non-clinical settings.

## Introduction

The concept of metacognition, which is one of the most important mental activities and leads to a number of mental disorders when it is deficient, was initially expressed as “metacognitions” by Flavell in the late 1970s and only started to be studied in Turkey towards the beginning of 2000s. Metacognition is a general term used to describe a set of interconnected psychological and neuropsychological abilities that enable people to understand their own and others’ mental states, beliefs, desires, intentions, actions and attitudes (Pedone et al., 2017; Semerari et al., 2003). There are various definitions of metacognition in the literature. In a broad definition, metacognition can be defined as understanding the behavior and attitude of the people who have different mental states, desires, beliefs, emotions and intentions. Individuals’ awareness of their own and others’ feelings and thoughts, capabilities of problem solving and coping skills, self-monitoring, self-evaluation and self-decentralisation and mastery are the units of metacognitive self-awareness skills (Norman et al., 2019). Although meta-cognition cannot be directly observed, it forms a basis for people’s behaviors, their reactions and predictions about the future (Howlin et al., 1999; Premack and Woodruff, 1978). The difference between cognition and metacognition is that cognition refers to the mental processes and activities involved in acquiring, processing, and using information and includes tasks such as thinking, perceiving, remembering, and problem-solving. In contrast, metacognition is the awareness and understanding of these cognitive processes. It involves thinking about and reflecting on how you think, monitor your own thought processes, and regulate your learning strategies. In other words, cognition is the actual thinking and processing of information, while metacognition is thinking about and managing that thinking process (Frith, 2012).

Researchers have studied metacognition within different theoretical perspectives (Pedone et al., 2017). One of the main term and related construct is theory of mind (Premack and Woodruff, 1978; Baron-Cohen, 1995; Frith and Frith, 2000). Theory of mind is an ability to understand and mentally represent the intentions, beliefs, desires, and knowledge of others. It is the ability to understand one’s own and others’ thoughts, intentions, desires, emotions and other internal states and to interprete individuals’ behavior (Baron-Cohen, 1995; Flavell, 1976; Fonagy, 1991; Frith and Frith, 2006; Frith and Happé, 1999; Wellman et al., 2001). Even if metacognition and theory of mind are used interchangeably, they have different aspects. Theory of mind is about understanding the mental states of oneself and others. It strengthens social skills through the development of healthy social relationships and successful communication. Metacognition enables individuals to understand and define their own cognitive processes. Individuals who are aware of their cognitive processes are more likely to be successful in learning, making judgments, strategic decisions, and applying functional problem-solving techniques (Dutemple et al., 2023). Metacognition and theory of mind have also been associated with mirror neurons in the orbitofrontal and lateral prefrontal cortex, which play a role in individuals’ understanding of the wishes, desires and intentions of the other person, empathy, and emotions (Rizzolatti et al., 1996; Fleming et al., 2012; Praszkier, 2016).

Pedone et al. (2017) based MSAS scale and the term metacognition in the Metacognitive Multifunctional Model (MMFM) (Semerari et al., 2003) which emphasises common factors underlying constructs such as mental contents and cognitive functions. MMFM views metacognition as a set of skills. This model focuses on the functional aspect of metacognition, which is (1) identifying and explaining mental states of oneself and others based on internal experience and observable behavior, (2) thinking and reasoning about various mental contents, such as mental states, (3) decision-making in complex, chaotic situations, problem solving and using mental information to cope with suffering (Carcione et al., 2010). There are two other possible approaches to metacognition which are the neuroscience and social cognition development. The first approach suggests that cognitive neuroscience is “the study of the mind through the brain” and reduces the psychological processes to neurological processes (Greene and Cohen, 2004). The shortcoming of this view is that neurology particularly deals with causes. Intending on something and acting on it requires a brain function and neurological processes need to be involved in the activity. However, intentions cannot be studied solely by examining the brain because they are always embedded in social situations (Hacker and Bennett, 2003; Racine and Carpendale, 2007). The other approach is the developmental system approach which is investigating the neurological causal preconditions for thinking about social issues and the ways in which social experience shapes neurological processes (Griffiths and Stotz, 2000). This approach is based on psychological concepts. The other approach is “the self-regulation executive function theory” which is developed by Wells and Matthews (1994, 1996). According to that theory, metacognitions are effective in the development and maintenance of psychopathologies and also in the awareness of various feelings, identifying and labeling the emotions, understanding the thoughts and predictions of the behaviors. So, metacognitions may also have negative effects on individuals’ psychological well-being by developing dysfunctional strategies (Flavell, 1976; Wells, 2000; Wells and Purdon, 1999).

To establish a comprehensive model of metacognition, Lysaker et al. (2018) have proposed integrated model of cognition that encompasses a spectrum of activities. At one end of this spectrum is the awareness of distinct mental experiences, such as specific thoughts, feelings, and desires. At the other end is the integration of these individual experiences into a larger, complex understanding of oneself and others. These two ends of the spectrum are interconnected, as one’s broader sense of self is shaped by individual experiences, and individual experiences are influenced by this broader understanding. According to this model, metacognitive processes are crucial for individuals to have a unified and coherent sense of self and others in any given moment. When these processes are functioning optimally, individuals can effectively engage in multiple mental tasks simultaneously and with ease. Metacognition can be seen as a broad concept that encompasses various cognitive functions, ranging from simple individual processes to more intricate and all-encompassing cognitive functions, which also take into account neurocognitive and social cognitive aspects (Fekete et al., 2022).

Interest in metacognition has grown widely in fields such as education, developmental psychology and cognitive sciences as people have realised that they do not solve problems by simply processing information. In particular, since the beginning of the twenty-first century, interest in the association between metacognition and mental illness has grown rapidly. Impairments in metacognition seen to be the consequence of symptoms of various mental disorders. Many studies in the literature have revealed that metacognition problems lead to various psychopathologies and neurodevelopmental disorders. The most notable among these are schizophrenia (Frith, 1992), borderline personality disorder (Bateman and Fonagy, 2006) and other personality disorders (Semerari et al., 2014), post-traumatic stress disorder, autism spectrum disorder, asperger syndrome, attention deficit hyperactivity disorder (Liotti and Prunetti, 2010).

Metacognition is the cause of prognosis of mental illnesses and also it effects the learning processes negatively. In addition to the lack of insight in individuals, the development of a healthy self-perception and perception of the other is interrupted due to the difficulties individuals have in understanding the feelings, thoughts and intentions of others (Dimaggio et al., 2007a,b, 2015). Furthermore, metacognitive deficits have been associated with poor social functioning (Bo et al., 2015), emotion regulation difficulties (Harder and Folke, 2012), and neurocognitive impairment in verbal and visual memory and processing speed (Nicolo et al., 2012). It is observed that individuals with developed metacognitive skills are able to maintain stable social relationships because they have adequate emotion regulation and social skills (Bender et al., 2011).

It is very difficult to measure metacognition, which is so effective in the social, psychological and academic development of individuals, but due to the effect of metacognition on individuals with clinical features, valid, reliable and fast measurement tools are needed. Discourse analysis, interviews, self-assessment scales, laboratory studies, questionnaires are used to measure metacognition (Pedone et al., 2017; Martiadis et al., 2023). Among these, discourse analysis and interview techniques have higher validity, but are considered to be more costly due to time limit and also they need training of the individuals. Performance tests are used for neuropsychological assessments. Due to the need for social acceptability, performance tests provide more reliable results than self-assessment scales (Lezak and Bowler, 2015).

One of the ways of assessing metacognition is the self-assessment method. MSAS provides a complementary source of data by directly self-assessing individuals’ thoughts and awareness about their cognitive processes in a fast and reliable way (Pedone et al., 2017). This scale was developed in Italian language. Faustino et al. (2019) was conducted Portuguese adaptation of the MSAS Fernández-Rubio et al. (2023) adapted MSAS into Spanish.

MSAS offers information about how individuals perceive their own thinking processes and insights which may not always align with what can be observed through external assessments or interviews and while interviews and performance tests can provide valuable insights into metacognition, they often capture observable behaviors and may not fully capture an individual’s metacognitive awareness or beliefs. The other most commonly used scales in studies on metacognition are the Thought Control Questionnaire (TCQ) (Reynolds and Wells, 1999), the Anxious Thoughts Inventory (AnTI) (Wells, 1994), the Metacognitions Questionnaire (MCQ-30) (Wells and Cartwright-Hatton, 2004) and The Metacognitive Awareness Inventory (MAI) (Schraw and Dennison, 1994). The first two of these scales can be used in both clinic and non-clinical populations. Metacognitive Self-Assessment Scale (MSAS) and the Metacognitive Awareness Inventory (MAI) are both used in the field of psychology and education to assess an individual’s metacognitive awareness and self-assessment abilities. Metacognitive Self-Assessment Scale focuses on assessing an individual’s ability to accurately self-assess their own metacognitive skills and strategies. It evaluates individuals’ awareness of their own metacognitive processes such as differentiation, integration, monitoring, and mastery. Besides, the Metacognitive Awareness Inventory (MAI) measures general metacognitive awareness. It evaluates individuals’ ability to understand and use metacognitive strategies across various cognitive tasks and domains. The Meta-Cognitions Questionnaire (MCQ-30) focuses on measuring metacognitive beliefs and thought patterns related to worry and rumination, often associated with anxiety and related disorders. The MCQ-30 is frequently used in clinical and research contexts to evaluate metacognitive beliefs that are thought to contribute to the development and maintenance of anxiety disorders. It enables clinicians and researchers to comprehend the cognitive processes underlying excessive worry and rumination. However, The MSAS can be used with a wider range of populations and also used for the assessment of metacognitive deficits during psychotherapy.

Since there is no measurement tool to assess the metacognition self awareness in Turkey, in this study, the Metacognition Self-Assessment Scale (MSAS) which is a preliminary, quick and simple screening tool for the assessment of metacognition was adopted into Turkish. MSAS Turkish version adaptation will provide a tool for multidimensional assessment of metacognition skills in Turkish culture. The aim of this study was to validate the MSAS test in a Turkish-speaking non-clinical population and compare the four-factor structure.

## Method

### Participants

The study involved 467 non-clinical participants, 316 of whom were females (67.7% of the sample) and 151 of whom were males (32.33%). Participants’ ages ranged from 18 to 31 years with an average age of 24.18 (±3.25). Data from 467 participants from the general population were analyzed. These participants were recruited from Beykoz University in Istanbul.

### Data collection tools

#### The metacognition self-assessment scale (MSAS)

The Metacognition Self-Assessment Scale (MSAS) is an 18-item self-report instrument developed to comprehensively measure five distinct sub-functions of metacognition, grounded in a functional-focused perspective model known as the Metacognitive Multifunction Model (MMFM). According to Pedone et al. (2017), the MSAS was developed to measure five metacognitive skills: (1) monitoring, (2) differentiation, (3) integration, (4) decentralisation and (5) mastery. These five capabilities are integrated into the following characteristics. (a) Reflection on one’s own mental states (self domain: understanding one’s own mind—UM), (b) reflecting on the mental states of domain: understanding the mind of others—UOM) and (c) coping with psychological pain and interpersonal problems (mastery—M). In the original study, the MSAS consists of four factor structures: (1) the first factor predicting monitoring and integration (self-reflexivity), (2) the second factor predicting differentiation and decentering, (3) the third factor predicting and measuring understanding the minds of others (self-other), and (4) the fourth factor including regulation and control abilities (mastery). MMFM represents an important aspect of metacognition specifically referring to a set of skills intended as a set of functions, rather than a singular ability. The MSAS is scored using a five-point Likert scale, ranging from 1 (never) to 5 (almost always), yielding a raw score spectrum of 18 to 90. Higher scores on the MSAS are indicative of enhanced self-evaluation of metacognitive abilities. The five targeted abilities within the MSAS include: (1) Self Reflectivity (F1), encompassing monitoring and integration of the Self, signifying one’s ability to recognise and articulate their own thoughts and emotions; (2) Critical Distance (F2), involving differentiation and decentration, highlighting critical comprehension and the discernment of thoughts’ subjectivity; (3) Mastery (F3), relating to individual strategies utilised in managing psychological and interpersonal problems, coping with psychological pain; and (4) Understanding Others’ Mind Monitoring (F4), focusing on the recognition of others’ mental states. A four-factor solution to the MSAS was found to account for 57% of the total variance, with each factor elucidating more than 5% of the unrotated variance. Cronbach alpha for the MSAS subscales ranged between 0.72 and 0.87, reflecting satisfactory internal consistency for all subscales and the overall metacognitive function as measured by the total MSAS score. Confirmatory factor analysis of a four-factors model for both samples showed that the Chi Square/df Ratio is consistent with a good fit (Sample 1, 14.83; Sample 2, 14.63). The NNFI, NFI, and CFI indices were above 0.90 for both samples, consistent with an adequate fit, as well as RMSEA (Sample 1, 0.064; Sample 2, 0.065). These statistical outcomes bear significant evidence supporting the theoretical suppositions concerning the organisation of MMFM functions. They signify that the MSAS can be perceived as comprising distinct and independent components that signify different metacognitive functions.

### Procedures

The process of translating the MSAS into Turkish involved a meticulous two-stage preliminary study. Initially, the scale items were translated from English to Turkish, allowing for multiple alternative translations for each item. These initial translations were then reviewed by seven psychology professionals in Turkey, all holding doctoral degrees and proficient in English. Each professional was tasked with selecting the most suitable translation for each item or proposing a new alternative where necessary. Through this expert evaluation process, the items that garnered consensus among the academicians were incorporated into the Turkish version of the scale. Subsequently, the back-translation stage was undertaken to ensure the accuracy and fidelity of the translated items. Three different experts proficient in both English and Turkish independently translated the finalized scale items back into English. Discrepancies or inconsistencies identified during this back-translation process were addressed through necessary adjustments to refine the Turkish version of the scale. This rigorous translation process, involving both forward and back-translation stages, was crucial in ensuring the linguistic and conceptual equivalence of the Turkish version of the MSAS. By engaging a panel of qualified professionals and employing systematic translation techniques, the study aimed to maintain the integrity and validity of the scale across different linguistic and cultural contexts. The final Turkish form of the MSAS was thus established through a comprehensive process of translation and validation, guided by the expertise and feedback of experienced professionals in the field of psychology and linguistic. Before the initiation of the study, the Beykoz University’s Ethical Committee granted ethical clearance. The compliance of the study with ethical principles was evaluated by the ethics committee of the institution where the study was conducted and ethical approval was obtained (31.01.2023/4). In the study, written informed consent was obtained from the sampled patients.

### Data analysis

In this study, construct validity was investigated using first-order and second-order Confirmatory Factor Analysis (CFA) conducted in RStudio, utilising the lavaan and semTools packages (Rosseel, 2012). Additionally, internal consistency reliability and discrimination coefficients were computed by comparing the 27% highest and 27% lowest groups, and a comparison between these two groups was performed using an Independent Samples *t*-test. Criteria validity was further assessed through the Pearson correlation coefficient, while the assessment of multivariate normality was conducted using the “MVN” package (Korkmaz et al., 2014; Mardia, 1970) in Rstudio (R Core Team, 2012).

## Results

### Prior analyses

Prior to embarking on the analyses for reliability and validity, preliminary descriptive statistics were computed. The skewness values were found to be distributed within the range of −1.149 to −0.276, whereas the kurtosis values fluctuated between −0.029 and 1.284. To scrutinise the multivariate normality of the data’s distribution, Mardia’s test for multivariate skewness and kurtosis was employed. The ensuing results yielded a Mardia’s Skewness value of 3214.33 and a Mardia’s Kurtosis value of 39.98, thereby signifying a deviation from the assumption of multivariate normality. Consequently, it was inferred that the data were not characterised by a normal distribution.

Given that multivariate normality is a desirable property for the utilisation of Maximum Likelihood estimation (as articulated by Kline, 1998), the present study elected to apply Robust Maximum Likelihood estimation as a corrective measure to contend with this issue of non-normality. This methodological adjustment allowed for the continuation of the study with appropriate statistical rigor, ensuring that the analyses were not compromised by the underlying distributional characteristics of the data.

### Construct validity-confirmatory factor analysis

To investigate the construct validity encompassing the 18 items and four underlying subscales, a first-order Confirmatory Factor Analysis (CFA) was undertaken. The ensuing fit indices present a comprehensive assessment of the model’s congruence with the empirical data: the ratio of the chi-square to degrees of freedom was 3.19 (*χ*^2^/df = 411.06/129), coupled with a *p*-value of 0.00; the Goodness of Fit Index (GFI) equaled 0.91; the Adjusted Goodness of Fit Index (AGFI) registered at 0.88; the Comparative Fit Index (CFI) was 0.91; the Standardised Root Mean Square Residual (SRMR) was found to be 0.054, and the Root Mean Square Error of Approximation (RMSEA) measured 0.068, bounded by a 95% Confidence Interval ranging from 0.061 to 0.076.

**Figure 1:**
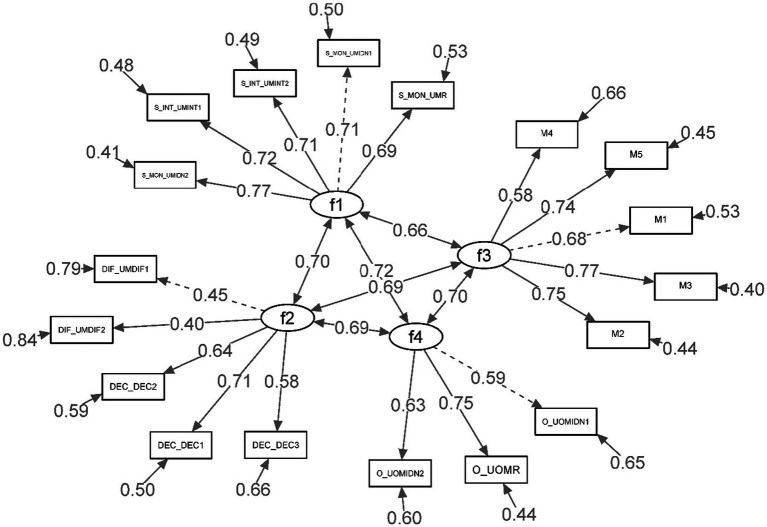
Standardized factor loadings, correlations among factors for the first order CFA.

Standardised factor loadings along with inter-factor correlations are succinctly illustrated in Figure 1.

Moreover, Table 1 systematically delineates the statistical attributes for each item, encompassing the standard errors, standardised coefficients, *t*-values, and corresponding *R*^2^.

**Table 1:** Standardised factor loadings, *t* values, standard errors and *R*^2^.

Items	Estimates	SE	*t*	*p*	95% CI lower	95% CI upper	Standardised factor loadings	*R* ^2^
Factor—1: Self MON INT								*0.70*
Self_MON_UMIDN1 (1A)							0.71	0.50
Self_MON_UMIDN2 (2A)	1.17	0.08	15.10	0.0000	1.02	1.32	0.77	0.59
Self_MON_UMREV1 (3A)	1.06	0.10	10.76	0.0000	0.87	1.26	0.69	0.47
Self_INT_UMINT1 (6A)	1.19	0.09	13.40	0.0000	1.02	1.37	0.72	0.52
Self_INT_UMINT2 (7A)	1.21	0.10	12.51	0.0000	1.02	1.40	0.71	0.51
Factor—2: DIF / DEC								*0.70*
DIF_UMDIF1 (4A)							0.45	0.21
DIF_UMDIF2 (5A)	0.91	0.11	8.33	0.0000	0.70	1.13	0.40	0.16
DEC_DEC1 (1C)	1.37	0.19	7.32	0.0000	1.01	1.74	0.71	0.50
DEC_DEC2 (2C)	1.23	0.17	7.03	0.0000	0.89	1.57	0.64	0.41
DEC_DEC3 (3C)	1.17	0.16	7.28	0.0000	0.85	1.48	0.58	0.34
Factor—3: Mastery								*0.66*
M1 (1D)							0.68	0.47
M2 (2D)	1.02	0.06	16.79	0.0000	0.90	1.14	0.75	0.56
M3 (3D)	1.06	0.07	14.37	0.0000	0.91	1.20	0.77	0.60
M4 (4D)	0.87	0.08	10.88	0.0000	0.71	1.03	0.58	0.34
M5 (5D)	1.01	0.07	14.38	0.0000	0.88	1.15	0.74	0.55
Factor—4: Other MON								*0.72*
Other_UOMIDN1 (1B)							0.59	0.35
Other_UOMIDN2 (2B)	1.07	0.42	2.51	0.0119	0.24	1.90	0.63	0.40
Other_UOMREV1 (3B)	1.25	0.41	3.03	0.0024	0.44	2.06	0.75	0.56

The standardised coefficients were observed to span from 0.40 to 0.77, with all factor loadings manifesting statistical significance at the 0.001 level. This substantiates robust associations between the observed variables and their pertinent latent factors.

The descriptive statistics and internal consistency reliabilities of the Metacognition Self-Assessment Scale (MSAS) factors were analysed in a sample of 467 participants (see Table 2). Alpha reliabilities for the four factors ranged from 0.68 to 0.84, demonstrating acceptable to strong internal consistency according to guidelines provided by Nunnally (1978). Specifically, Factor 1 and Factor 3 manifested strong reliability with alpha values of 0.84 and 0.83, respectively, while Factor 2 and Factor 4 exhibited acceptable reliability with alpha values of 0.69 and 0.68, respectively.

**Table 2:** Descriptive statistics, internal consistency reliability and correlations among the MSAS factors (sample *n* = 467).

	Mean	SD	Alpha	F1	F2	F3	F4
Factor 1	4.19	0.59	0.84	–			
Factor 2	4.11	0.56	0.69	0.70* (0.54*)	–		
Factor 3	3.76	0.67	0.83	0.66* (0.54*)	0.69* (0.52*)	–	
Factor 4	4.04	0.59	0.68	0.72* (0.58*)	0.69* (0.47*)	0.70* (0.53*)	–

*p < 0.01. The correlation coefficients in parentheses represent correlations among the total scores of the factors, whereas those outside of the parentheses represent correlations among the latent factors.

The means for the four factors were situated in the range of 3.76 to 4.19, and standard deviations ranged between 0.56 and 0.67, indicating a relatively concentrated distribution of scores around the mean values. These mean scores highlight a generally high level of agreement with the statements presented in various MSAS factors.

Furthermore, correlations among the factors were examined both at the latent level and using total scores. All correlations were significant at the *p* < 0.01 level, underscoring the presence of significant associations between different factors of the MSAS. The correlation coefficients derived from the latent variables were slightly higher in magnitude compared to those derived from total scores, delineating more pronounced relationships at the conceptual level underlying each factor.

Subsequent to the execution of the first-order Confirmatory Factor Analysis (CFA), a second-order CFA was conducted to further evaluate the structure of the scale. The ensuing goodness of fit indices demonstrated results as follows: *χ*^2^/df (412.64/131) = 3.15, *p* = 0.00, Goodness of Fit Index (GFI) = 0.91, Adjusted Goodness of Fit Index (AGFI) = 0.88, Comparative Fit Index (CFI) = 0.91, Standardised Root Mean Square Residual (SRMR) = 0.055, and Root Mean Square Error of Approximation (RMSEA) = 0.068 (within a 95% Confidence Interval for RMSEA ranging from 0.061 to 0.075).

**Figure 2:**
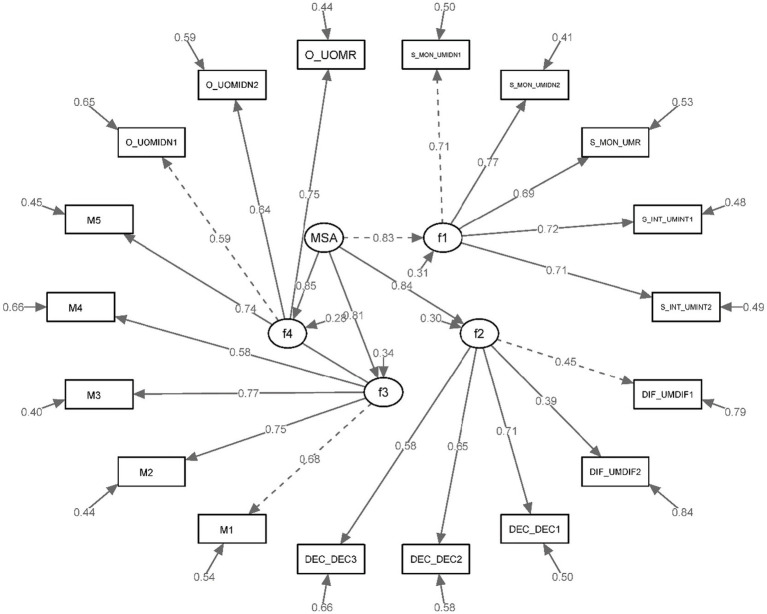
Standardized factor loadings for the second-order CFA.

These metrics provide substantial empirical support for the proposed scale structure, mirroring the congruence found in the first-order analysis. The model was corroborated to fit the data soundly. Standardised factor loadings and inter-factor correlations, encapsulating the intricacies of the relationships among variables, are illustrated in Figure 2.

These goodness of fit results indicate substantial support for the proposed scale structure, and the model exhibited a good fit with the data as second-order analysis. These results suggest robust connections between the observed variables and their respective latent factors.

### Construct validity-total-item correlation and internal consistency

Total-item correlation is a statistical method frequently employed in the nascent stages of scale development to investigate the relationship between each individual item and the aggregate score of the scale. It serves as an indicative measure to understand how well an individual item corresponds with the overarching construct it is intended to assess. Table 3 delineates the total-item correlations and Cronbach’s Alpha coefficients, providing insights into internal consistency and reliability of the scale. Cronbach’s Alpha coefficient for the total score of the scale is 0.89. In summary, the overall scale meets acceptable standards, as outlined by Tabachnick and Fidell (2013). This lends support to the validity and reliability of the measurement instrument, reflecting its coherence in measuring the intended constructs.

**Table 3:** Item-total correlations.

Factors and items	Item-total correlations
Factor—1: Self MON INT	
Self_MON_UMIDN1 (1A)	0.64
Self_MON_UMIDN2 (2A)	0.70
Self_MON_UMREV1 (3A)	0.61
Self_INT_UMINT1 (6A)	0.65
Self_INT_UMINT2 (7A)	0.64
Factor—2: DIF / DEC	
DIF_UMDIF1 (4A)	0.41
DIF_UMDIF2 (5A)	0.38
DEC_DEC1 (1C)	0.50
DEC_DEC2 (2C)	0.50
DEC_DEC3 (3C)	0.45
Factor—3: Mastery	
M1 (1D)	0.61
M2 (2D)	0.65
M3 (3D)	0.67
M4 (4D)	0.54
M5 (5D)	0.67
Factor—4: Other MON	
Other_UOMIDN1 (1B)	0.36
Other_UOMIDN2 (2B)	0.54
Other_UOMREV1 (3B)	0.59

### Test–retest reliability

The reliability of scale was investigated by test re-test and for evaluating the test–retest reliability of the scale, 100 participants are included in the search. Significant positive correlations were observed across all subscales and the general scale. Specifically, the test–retest reliability for Subscale A was *r*(90) = 0.80, *p* < 0.01, Subscale B was *r*(90) = 0.64, *p* < 0.01, Subscale C was *r*(90) = 0.68, *p* < 0.01, Subscale D was *r*(90) = 0.75, *p* < 0.01, and for the general scale was *r*(90) = 0.88, *p* < 0.01. Similar significant correlations were observed between the other subscales, indicating good reliability of the scale.

### Measurement invariance

A series of multigroup analyses were systematically conducted to assess configural, metric, scalar, and strict invariance models, successively comparing the fits between male and female groups, as well as younger and older adult groups. Leveraging the configural model as a benchmark for subsequent equivalence model evaluations as delineated by Byrne (2010), it was discerned that all pertinent fit indices upheld the robustness of the four-factor structure elucidated in Table 4. This underscores the resonance of the model across diverse demographic delineations, showcasing its universal applicability and validity both in terms of gender and age brackets.

**Table 4:** Model comparisons for invariance across sex and age.

Model	χ^2^	*df*	RMSEA	SRMR	CFI	(ΔCFI)
Sex group measurement models						
Configural	623.94	258	0.078	0.062	0.8867	
Metric	636.31	272	0.076	0.063	0.8872	0.0005
Scalar	647.10	286	0.074	0.063	0.8882	0.0010
Strict	669.86	304	0.072	0.066	0.8867	−0.0015
Age group measurement models						
Configural	584.64	258	0.074	0.061	0.8993	
Metric	622.35	272	0.074	0.064	0.8920	−0.0073
Scalar	646.99	286	0.074	0.066	0.8887	−0.0033
Strict	700.90	304	0.075	0.067	0.8777	−0.0111

According to Cheung and Rensvold (2002) ΔCFI < 0.01 implies that the invariance assumption holds.

#### Item discrimination ratio

In accordance with Cureton’s (1957) methodology, item discrimination coefficients were ascertained in the present study. The procedure involves the computation of the Item Discrimination Ratio, utilising a critical ratio of 27%, which is extracted from the standard normal distribution curve of errors. To carry out the analysis, scores were segregated into the lower and upper 27% categories for each underlying factor, enabling a comparative evaluation of individual items in relation to these delineated groups. Table 5 encapsulates the findings derived from this analytical process.

**Table 5:** Discrimination coefficients, independent *t* test results.

Dependent variable	*t*	*df*	*p*	*d*	95% CI
Factor—1: Self MON INT					
Self_MON_UMIDN1 (1A)	−13.40	188.62	< 0.001	−1.69	[−1.98, −1.40]
Self_MON_UMIDN2 (2A)	−14.48	211.67	< 0.001	−1.82	[−2.12, −1.53]
Self_MON_UMREV1 (3A)	−11.77	208.98	< 0.001	−1.48	[−1.76, −1.20]
Self_INT_UMINT1 (6A)	−14.77	218.96	< 0.001	−1.86	[−2.16, −1.56]
Self_INT_UMINT2 (7A)	−15.34	197.43	< 0.001	−1.93	[−2.23, −1.63]
Factor—2: DIF / DEC					
DIF_UMDIF1 (4A)	−9.33	233.83	< 0.001	−1.18	[−1.44, −0.91]
DIF_UMDIF2 (5A)	−8.96	234.79	< 0.001	−1.13	[−1.39, −0.86]
DEC_DEC1 (1C)	−13.11	212.11	< 0.001	−1.65	[−1.94, −1.36]
DEC_DEC2 (2C)	−10.63	205.03	< 0.001	−1.34	[−1.61, −1.06]
DEC_DEC3 (3C)	−12.28	191.41	< 0.001	−1.55	[−1.83, −1.26]
Factor—3: Mastery					
M1 (1D)	−16.07	244.06	< 0.001	−2.02	[−2.33, −1.72]
M2 (2D)	−16.11	207.37	< 0.001	−2.03	[−2.64, −1.72]
M3 (3D)	−14.74	229.57	< 0.001	−1.86	[−2.15, −1.56]
M4 (4D)	−10.81	249.28	< 0.001	−1.36	[−1.64, −1.09]
M5 (5D)	−14.06	225.23	< 0.001	−1.77	[−2.06, −1.48]
Factor—4: Other MON					
Other_UOMIDN1 (1B)	−13.25	186.86	< 0.001	−1.67	[−1.96, −1.38]
Other_UOMIDN2 (2B)	−10.36	232.23	< 0.001	−1.30	[−1.58, −1.03]
Other_UOMREV1 (3B)	−14.20	237.73	< 0.001	−1.79	[−2.08, −1.49]

The findings revealed that every individual item adeptly differentiated between the upper and lower scoring groups. Significantly, the *t*-test outcomes for each item were found to be statistically significant, underscoring the robust discrimination capability of the items.

## Discussion

In this study, we conducted the Turkish adaptation of the Metacognition Self-Assessment Scale, and assessed the psychometric properties of the measurement tool.

When the research group of the Turkish version of the scale is compared with the participants of the original and other versions, it can be stated that they are in the same age groups as the Portuguese group (Faustino et al., 2019) since the average age is 32. The age group of the original version is older, while the Spanish version is younger. In the development and all adaptation studies of the scale, the proportion of women was significantly higher in the research group.

The original scale Cronbach alpha ranged between 0.72 and 0.87. Besides, The Turkish scale Cronbach alpha is between 0.68 and 0.89. In the Spanish version, it ranged between 0.65 and 0.83, and in the Portuguese version, between 0.73 and 0.88. The evaluation of internal consistency for both the four subscales and the overall scale meets acceptable standards, as outlined by Tabachnick and Fidell (2013). The results of the Turkish adaptation of MSAS scale suggest that the measurement tool is also found reliable instrument. In the MSAS score reliability study, internal consistency data were similar to the original, the Portuguese (Faustino et al., 2019) and the Spanish studies (Fernández-Rubio et al., 2023).To investigate the construct validity encompassing the 18 items and four underlying subscales, a first-order Confirmatory Factor Analysis (CFA) was undertaken. Confirmatory factor analysis (CFA) was conducted to determine whether the factor structure of the original form would be confirmed in a sample of 467 Turkish individuals. Considering the limits of the fit index for CFA (Hu and Bentler, 1999), the model was found to be compatible. Considering that the reliability level for measurement tools that can be used in research is 0.70 (Tezbaşaran, 1996), it can be stated that the scale has a satisfactory level of reliability. According to the findings obtained from the validity and reliability studies, it can be stated that the scale is ready for use. Subsequent to the execution of the first-order Confirmatory Factor Analysis (CFA), a second-order CFA was conducted to further evaluate the structure of the scale. The original scale NNFI, NFI, CFI indices are above 0.90. RMSEA value is between 0.064 and 0.065. In this study, Turkish version of MSAS scale GFI, CFI values are above 0.91. RMSEA value is 0.068. The ensuing goodness of fit indices demonstrated results as adequate. These indices collectively render substantial empirical endorsement for the hypothesised scale configuration. The model’s alignment with the observed data can be classified as good, demonstrating robust construct validity. These goodness of fit results indicate substantial support for the proposed scale structure and the model exhibited a good fit with the data as second-order analysis. These results suggest robust connections between the observed variables and their respective latent factors. The evidence concerning internal structure was comparable to the initial version of MSAS, as well as the Portuguese and Spanish versions with fit indices meeting the acceptable standards.

The correlations among the total scores of the factors of the scale were between 0.53 and 0.58 in our study. In the original version, it is between 0.26 and 0.40. In the Portuguese version, it is between 0.43 and 0.60. Factor 4, understanding other minds, showed low correlations with other sub-dimensions in the Spanish and Portuguese versions, whereas in our study its correlations with other factors were 0.72, 0.69 and 0.70, respectively. Therefore, it can be stated that the sub-dimensions have a higher correlation with each other in the Turkish version. Factor correlations were ascertained and denoted noteworthy relationships between various factors. The statistical delineation revealed a pronounced and statistically significant correlation. In The Turkish adaptation scale, it was found significant correlations between 4 Factors. These results not only corroborate the interrelations between the specified factors but also reinforce the validity and consistency of the constructs.

When the measurement invariance results of our study are analysed, it reveals its universal applicability and validity in terms of both gender and age groups. This finding is in line with the Spanish version (Fernández-Rubio et al., 2023). To determine the criterion validity of the scale, Beck Cognitive Insight Scale (BCIS), Eysenck Personality Inventory (EPI), Moritz Control Scale were used in the Spanish adaptation. In the Portuguese adaptation study, Interpersonal Reactivity Index (IRI), Cognitive Fusion Questionnaire (CFQ) (Faustino et al., 2019) were used. In this study, a scale was not used due to the lack of Turkish versions of this scale and similar ones. The Thought Control Questionnaire (TCQ) (Wells and Davies, 1994), the Anxious Thoughts Inventory (AnTI) (Wells, 1994), and the Metacognitions Questionnaire (MCQ-30) (Wells and Cartwright-Hatton, 2004) are the three most often used measures in metacognition research (Wells and Cartwright-Hatton, 2004). The first two of these measures can be applied to both psychopathological and normal populations. However, the Metacognition Questionnaire (MCQ-30) was developed using metacognitive concepts linked to psychopathologies and is therefore appropriate for testing metacognitions in psychopathologies (Irak and Tosun, 2008) According to Wells and Cartwright-Hatton (2004), people with generalized anxiety disorder differ from those with other anxiety disorders in terms of negative views about thoughts. Metacognitive characteristics evaluated by the MCQ-30 are positively connected to obsessive-compulsive symptoms, according to studies (Gwilliam et al., 2004; Hermans et al., 2003; Janeck et al., 2003; Wells and Papageorgiou, 1998). Wells and Papageorgiou (1998) discovered that metacognitions were positively connected to pathological anxiety symptoms. The purpose of the MCQ-30 scale is to look into the connection between developing psychopatologies and metacognitions. The MCQ-30 scale, although adapted for use in Turkish, was not utilized because the construct it assesses is not equivalent to that of the MSAS scale. The MSAS is a practical self-report style scale that is easy to administer and assess. In this study, which aimed to examine the psychometric properties of the scale in Turkish individuals, similar results were obtained with the original scale. Confirmatory factor analysis results showed that the scale was parallel to its original form in terms of construct validity. The findings of the validity and reliability study revealed that the scale has sufficient validity and reliability to measure metacognitive self-evaluation skills. In conclusion, considering the values obtained from the adaptation study of MSAS into Turkish, it can be said that the scale is a valid and reliable measurement tool that can be used in Turkish culture to assess metacognitive self-awareness skills.

### Limitations

There are some limitations of this research. The original version of the MSAS scale, as well as the Spanish and Portuguese versions, utilized the general population as the sample. Pedone et al. (2017) who developed the scale noted that the study’s limitation was the lack of clinical sample participation. Similarly, our study also did not involve a clinical sample. Establishing future studies that incorporate a clinical sample will improve the scale’s applicability and enhance the strength and applicability of the Turkish version of the Metacognitive Self-Assessment Scale. Secondly, we were unable to perform criterion validity due to the absence of a metacognition scale in Turkish with a similar structure to the one we adapted. This is considered a limitation. Another limitation of our study is that it was conducted with a sample of young adults. Future research should consider expanding the age range of participants to assess the suitability of the MSAS across various life stages. Another limitation is that cultural factors were not considered as a variable in this study. Conducting qualitative studies to investigate these cultural factors will contribute to the scale’s refinement and ensure an accurate representation of the metacognitive process within the Turkish context.

The data that support the findings of this study are openly available in figshare at https://figshare.com/s/7a5a6464620411fe3877.

## Data Availability

The datasets presented in this study can be found in online repositories. The datasets can be found at: https://dataverse.harvard.edu/dataset.xhtml?persistentId=doi:10.7910/DVN/LUHWY0.
