# Supplementary material for: Psychometric properties and validation of the metacognitive self-assessment scale (MSAS) in a Turkish sample
Source: Front Psychol. 2024 Dec 12;15:1411733. doi: 10.3389/fpsyg.2024.1411733 (PMC11670667; doi:10.3389/fpsyg.2024.1411733)
Supplement: Supplementary file 2 [file Data_Sheet_2.docx]

**ÜST BİLİŞ ÖZ DEĞERLENDİRME ÖLÇEĞİ**

Aşağıdaki anket, insanların düşüncelerini, duygularını ve dahil oldukları sosyal ilişkileri tanımlama ve tarif edebilme becerileri hakkında ne düşündüklerini ele almaktadır.

Aşağıda sıralanan ifadeleri takip ederek, bunların sizi nasıl tanımladığına ilişkin düşüncenizi belirtebilirsiniz. Lütfen çarpı işareti koyarak size uygun ifadeyi yanıtlayınız.

| A | **Kendimle ilgili…** | Asla | Nadiren | Bazen | Sıklıkla | Neredeyse her zaman |
| --- | --- | --- | --- | --- | --- | --- |
| 1. | Kendi zihinsel yeteneklerimin (örneğin; hatırlama, hayal etme, fanteziler kurma, rüya görme, arzulama, karar verme, öngörme ve düşünme) farkına varabilir ve ayırt edebilirim. | 1 | 2 | 3 | 4 | 5 |
| 2. | Kendi duygularımı tanımlayabilir, ayırt edebilir ve adlandırabilirim. | 1 | 2 | 3 | 4 | 5 |
| 3. | Davranışlarıma yön veren düşüncelerin veya duyguların neler olduğunun farkındayım. | 1 | 2 | 3 | 4 | 5 |
| 4. | Kendimle ilgili fikirlerimin her zaman doğru olmayabileceğinin ve değişebileceğinin farkındayım. | 1 | 2 | 3 | 4 | 5 |
| 5. | İstediğim ya da umduğum şeylerin her zaman gerçekleşmeyebileceğinin ve olayları etkileme gücümün sınırlı olduğunun farkındayım. | 1 | 2 | 3 | 4 | 5 |
| 6. | Düşüncelerimi, duygularımı ve içinde bulunduğum ilişkileri net bir şekilde algılayabilir ve tanımlayabilirim. | 1 | 2 | 3 | 4 | 5 |
| 7. | Bir andan diğerine farklılık gösterseler bile düşüncelerim ve duygularım arasındaki bağı tanımlayabilirim. | 1 | 2 | 3 | 4 | 5 |

| B | **Başkalarıyla ilgili…** | Asla | Nadiren | Bazen | Sıklıkla | Neredeyse her zaman |
| --- | --- | --- | --- | --- | --- | --- |
| 1. | Hatırlama, hayal etme, fanteziler kurma, rüya görme, arzulama, karar verme, öngörüde bulunma ve düşünme gibi farklı zihinsel faaliyetleri anlayabilir ve ayırt edebilirim. | 1 | 2 | 3 | 4 | 5 |
| 2. | Tanıdığım insanların duygularını tanımlayabilir ve anlayabilirim. | 1 | 2 | 3 | 4 | 5 |
| 3. | Bir andan diğerine farklılık gösterseler bile tanıdığım insanların düşünceleri ve duyguları arasındaki bağı tanımlayabilirim. | 1 | 2 | 3 | 4 | 5 |

| C | **Kendini başkasının yerine koyabilme…** | Asla | Nadiren | Bazen | Sıklıkla | Neredeyse her zaman |
| --- | --- | --- | --- | --- | --- | --- |
| 1. | Başkalarının duygu, düşünce ve hislerinin merkezinde olmadığımın, diğer kişilerin benimle olan ilişkilerindeki davranışlarının; benim bakış açım ve ilişkimizden kaynaklanmayabileceğinin, kişilerin kendi düşünce ve hedefleri kaynaklı olabileceğinin farkındayım. | 1 | 2 | 3 | 4 | 5 |
| 2. | Başkalarının gerçekleri ve olayları benden farklı algılayabileceklerinin ve farklı yorumlayabileceklerinin farkındayım. | 1 | 2 | 3 | 4 | 5 |
| 3. | Yaşın ve yaşam deneyiminin başkalarının düşüncelerini, duygularını ve davranışlarını etkileyebileceğinin farkındayım. | 1 | 2 | 3 | 4 | 5 |

| D | **Sorun çözme…** | Asla | Nadiren | Bazen | Sıklıkla | Neredeyse her zaman |
| --- | --- | --- | --- | --- | --- | --- |
| 1. | Bir problemle başa çıkmak için kendimdeki bir davranışı engelleyebilir veya değiştirebilirim | 1 | 2 | 3 | 4 | 5 |
| 2. | Karşıma çıkan problemlerle kendi zihnimde çözümler üreterek başa çıkabilirim. | 1 | 2 | 3 | 4 | 5 |
| 3. | Görüşlerime ve inançlarıma meydan okuyan veya onları zenginleştiren/zenginleştirmeye çalışan sorunlarla başa çıkabilirim. | 1 | 2 | 3 | 4 | 5 |
| 4. | Sorunlar, başkalarıyla olan ilişkilerimle ilgili olduğunda bunları bu kişilerin kendi düşünce ve inançlarından kaynaklandığına inanarak çözmeye çalışırım. | 1 | 2 | 3 | 4 | 5 |
| 5. | Kendimi yönetme ve olayları etkileme konusundaki sınırlılıklarımı fark edip kabul ederek sorunlarla başa çıkabilirim. | 1 | 2 | 3 | 4 | 5 |
